# A Service Evaluation of Antenatal Detection of Small-for-Gestational-Age Infants in a UK National Health Service Trust

**DOI:** 10.7759/cureus.94901

**Published:** 2025-10-19

**Authors:** Harun H Ali, Tharanya Thiruchelvam, Tulika Singh

**Affiliations:** 1 School of Medicine, University of Leeds, Leeds, GBR; 2 Obstetrics - Fetal Medicine, Mid Yorkshire Hospitals NHS Trust, Wakefield, GBR

**Keywords:** antenatal screening, badgernet, customised growth centiles, fetal growth restriction, growth assessment protocol, maternal smoking, perinatal outcomes, small for gestational age, stillbirth prevention, symphyseal fundal height

## Abstract

Introduction

Infants with a birthweight below the 10th centile are classified as small-for-gestational age (SGA). SGA is closely associated with fetal growth restriction (FGR), and both represent significant risk factors for stillbirth. The UK continues to report comparatively high stillbirth rates, and antenatal detection of SGA is a recognised strategy for reducing this risk. This service evaluation assessed the antenatal detection rate of SGA infants at Mid Yorkshire Hospitals NHS Trust (MYHT).

Methods

This was a retrospective observational cohort service evaluation conducted between January and March 2020 using total-population sampling of all eligible cases. Using the BadgerNet maternity system, 124 SGA infants were identified, of which 110 were included after applying eligibility criteria. Data were collected on 12 potential risk factors, including maternal age, medical comorbidities, and history of SGA births. The overall detection rate for the Trust was calculated, and a multivariable (binary) logistic regression was performed using IBM SPSS Statistics for Windows, Version 26 (Released 2019; IBM Corp., Armonk, New York, United States) following descriptive analysis in Microsoft Excel. Missing data were addressed using multiple imputation, and supplementary analyses were conducted for contextual understanding.

Results

A total of 42.7% of SGA infants were detected in the antenatal period; 97.9% of these were identified following referral for an abnormal symphyseal fundal height (SFH) measurement. The regression model was statistically significant, χ²(12) = 47.7-50.9, p < 0.001. Maternal smoking was associated with a 3.9-fold increase in detection (95% CI, 1.01-14.9), while referral after an abnormal SFH measurement increased the likelihood of detection by 154-fold (95% CI, 14.9-1603.4).

Conclusions

The antenatal detection rate of SGA infants at MYHT mirrors the national average, yet most cases remain undetected across the UK. Detection was primarily driven by referral after abnormal SFH measurements, underscoring the value of routine screening. Maternal smoking also independently increased detection, reflecting its strong association with fetal growth restriction. National adoption of customised centiles in systems such as BadgerNet may improve alignment with clinical practice, support earlier recognition of at-risk pregnancies, and contribute to reducing stillbirth rates.

## Introduction

Small-for-gestational age (SGA) describes infants with a birthweight below the 10th centile for gestational age [1,2]. In the literature, SGA is often incorrectly interchanged with fetal growth restriction (FGR), which refers to fetuses that fail to reach their growth potential due to reduced growth velocity during pregnancy [2].

Although the definitions overlap, the terms are not synonymous. Up to 70% of SGA fetuses are considered “constitutionally small,” meaning their growth is appropriate when maternal size, ethnicity, and other factors are taken into account [3]. The remaining SGA fetuses are pathologically small due to causes such as placental insufficiency, chromosomal abnormalities, structural anomalies, or congenital infection [3]. Some of these cases also meet criteria for FGR.

The stillbirth rate in England and Wales is approximately 4 per 1,000 births, which remains higher than in many comparable high-income countries [4,5]. This issue is clinically significant because SGA and FGR are major risk factors for stillbirth, perinatal morbidity, and neonatal admission [6]. Although constitutionally small SGA fetuses have a modestly increased risk, most of the excess morbidity and mortality occurs in infants who are small due to FGR [3]. The only proven management strategy for SGA/FGR is timely delivery; therefore, accurate estimation of fetal weight and monitoring of growth velocity are essential. This enables clinicians to balance the risks of prematurity against those of intrauterine fetal death [3,7].

The definition of abnormal fetal size or growth is debated. The Royal College of Obstetricians and Gynaecologists (RCOG) advocates the use of customised growth charts, which incorporate maternal factors such as ethnicity and body habitus [3]. These charts help distinguish constitutionally small but healthy infants from those who are pathologically small, while also avoiding misclassification of appropriate-for-gestational age (AGA) or even large-for-gestational age (LGA) infants who appear small on population-based centiles [8,9]. In doing so, customised charts aim to reduce unnecessary interventions while optimising care for truly growth-restricted fetuses.

Fetal size is commonly assessed using symphyseal fundal height (SFH), measured from the uterine fundus to the pubic symphysis [10]. Classically, growth of 1 cm per week is considered normal, but in practice, measurements are plotted on charts to identify abnormal patterns. SFH is less reliable in high-risk pregnancies; in such cases, ultrasound-based fetal biometry, including abdominal circumference (AC), head circumference (HC), femur length (FL), and biparietal diameter (BPD), provides more accurate weight estimation [3].

In the UK, routine antenatal screening includes assessment of risk factors for SGA/FGR such as advanced maternal age, smoking, prior SGA birth, and prior stillbirth [3,11]. RCOG guidelines recommend serial SFH measurements from 24 weeks in low-risk pregnancies, with referral for obstetric review and ultrasound if abnormalities are detected. For women with three or more minor risk factors, uterine artery Doppler assessment is recommended at 20-24 weeks. Those with major risk factors or abnormal uterine artery Doppler findings should undergo umbilical artery Dopplers and serial ultrasound measurements of estimated fetal weight (EFW) [3].

Despite these strategies, routine screening detects fewer than 25% of SGA cases [12]. To address this, the Perinatal Institute developed the Growth Assessment Protocol (GAP), which combines customised gestation-related optimal weight (GROW) charts with audit tools and clinical management protocols [12,13]. GAP has now been adopted by more than 70% of NHS Trusts, including Mid Yorkshire Hospitals NHS Trust (MYHT) [14].

The aim of this service evaluation was to assess the antenatal detection rate of SGA infants at MYHT. The primary objective was to estimate the proportion of SGA infants detected during the antenatal period using population-based centiles within the BadgerNet maternity system. Secondary objectives were to identify maternal and clinical predictors associated with antenatal detection using multivariable logistic regression and to compare population-based centiles with customised GROW centiles to evaluate classification differences.

## Materials and methods

Study design

This service evaluation employed a retrospective observational cohort design with quantitative methodology and was conducted at MYHT, an NHS organisation in West Yorkshire, England, whose main district general hospital, Pinderfields, provides maternity and neonatal services. The retrospective design provided access to pre-existing clinical data during the study period and enabled evaluation of antenatal detection rates of SGA babies and associated maternal factors. No formal study budget was required, as the evaluation was internally supported by MYHT and conducted using existing electronic health records.

Study population

**Figure 1:**
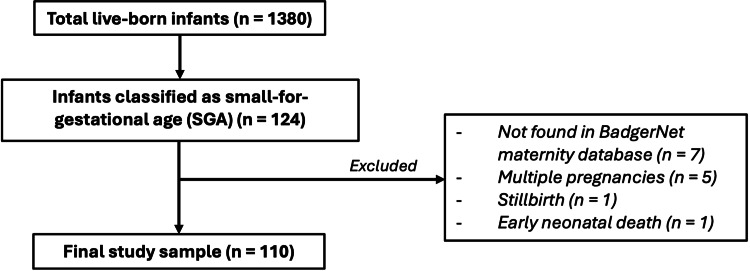
Study flow diagram of case selection and exclusions. The diagram outlines the selection of the study population from 1,380 total births. Of 124 infants classified as small-for-gestational age (SGA), 14 were excluded for specific reasons, resulting in a final study sample of 110 infants.

We reviewed all live-born infants delivered after 24 weeks’ gestation at MYHT between January 1, 2020, and March 31, 2020. Infants were included if they were classified as SGA according to BadgerNet automated population-based centiles. Exclusion criteria were multiple pregnancies (n = 5), stillbirths (n = 1), early neonatal deaths (n = 1), and cases not traceable in BadgerNet (n = 7). Multiple pregnancies were excluded because these infants are generally smaller and plotted on separate growth charts [15]. Stillbirths were excluded because they require separate methodological considerations, which were beyond the scope of this service evaluation. A total-population sampling approach was used, including all eligible cases during the study period. As the entire eligible population was evaluated, no formal sample size calculation was required. The full selection process is summarised in Figure 1, which shows the final study sample of 110 infants.

Data collection

Data were obtained from the BadgerNet Maternity system, an electronic maternity database. Variables collected included birthweight, mode of delivery, onset of labour, maternal age, booking body mass index (BMI), pregnancy screening results, aspirin use, pregnancy-associated plasma protein-A (PAPP-A) values, medical co-morbidities, history of SGA, smoking status (recorded as a binary yes/no variable), ethnicity, gravidity, parity, and adverse neonatal outcomes (including death). The MYHT data collection team provided a list of all eligible cases, after which two independent investigators manually extracted and cross-checked the relevant variables.

Data analysis

Descriptive analysis was performed using Microsoft Excel to generate summary tables, charts, and graphs. Categorical variables were presented as frequencies and percentages, and continuous variables were summarised using mean and standard deviation (SD) where applicable. The primary outcome was antenatal suspicion of SGA. Suspicion was defined as either referral after abnormal SFH measurement or identification on EFW scans. An abnormal SFH measurement was defined, in line with Trust protocol, as any value plotting below the 10th customised centile or showing static or reduced growth on serial measurements.

For secondary analyses, a multivariable (binary) logistic regression was conducted using IBM SPSS Statistics for Windows, Version 26 (Released 2019; IBM Corp., Armonk, New York, United States) to assess the association between SGA detection and maternal age, booking BMI, trisomy screening risk, PAPP-A, previous SGA, smoking status, gravidity, parity, history of miscarriage, SFH referral, hypertension, and diabetes. Odds ratios (ORs) with 95% confidence intervals (CIs) and p-values were calculated, with statistical significance set at p < 0.05. Assumptions for logistic regression were tested, including a dichotomous outcome variable, adequate events per variable [16], independence of observations, linearity, multicollinearity, and absence of significant outliers.

Missing data (0-16.4% across variables) were handled using multiple imputation in SPSS with default settings. Ten imputations were generated using the Mersenne Twister pseudo-random number generator (10 iterations per imputation). Type-appropriate models were used for all variables and the final imputation model included antenatal detection rates (binary outcome) as well as all predictors used in the final analyses to preserve inter-variable associations.

Supplementary analysis

To provide additional context, raw data on maternal BMI, parity, gestation, birthweight, and ethnicity were entered into GROW customised centile calculators. Infants classified as SGA by gestation-related optimal weight (GROW) charts were compared with those classified as SGA by BadgerNet centiles. Linear regressions were performed to assess the association between maternal smoking and customised centiles, and between customised centiles and referral after abnormal SFH.

Ethics and information governance

This study was conducted in line with University of Leeds ethics protocols. No high-risk ethical concerns were identified, and no new interventions were introduced; therefore, formal University ethics approval was not required. The National Health Service (NHS) Health Research Authority decision tool confirmed that the project constituted a service evaluation rather than research, and NHS Research Ethics Committee (REC) review was not required. Formal departmental approval was obtained from the service improvement lead at MYHT. All patient data were pseudonymised. Electronic files were password-protected, stored only on encrypted devices, and transferred exclusively through the secure NHS mail service and no paper records were created or retained.

## Results

Study population and antenatal detection rate

Between January 1 and March 31, 2020, there were 1,380 live births at MYHT. Of these, 124 infants had a birthweight below the 10th centile. After applying exclusion criteria, 110 infants remained in the study cohort and among these, 47 (42.7%) were suspected antenatally to be SGA. Almost all suspected cases, 97.9% were referred following abnormal SFH measurements; only one case was identified directly by ultrasound without prior referral. In total, 71 infants (64.5%) were referred due to abnormal SFH, of whom 46 (64.8%) were subsequently confirmed to be SGA on ultrasound.

Logistic regression

All assumptions for logistic regression were evaluated prior to analysis. Linearity was confirmed using the Box-Tidwell test, which showed non-significance of log-transformed interaction terms. Multicollinearity was identified for gravidity, parity, trisomy screening risk, and pregnancy-associated plasma protein-A (PAPP-A), with variance inflation factors (VIFs) of 9.26, 5.11, 3.61, and 3.37, respectively. To address this, gravidity and PAPP-A were excluded, lowering VIFs to between 1.084 and 1.778.

The initial model included 12 independent variables, which violated the requirement for at least 10 events per variable. Miscarriage history, not listed in the Royal College of Obstetricians and Gynaecologists (RCOG) risk assessment, was therefore removed. Outlier testing demonstrated no undue influence with standardised residuals ranging from -2.845 to 3.010, below the ±3.29 threshold [17,18]. The final model was statistically significant across the original and all 10 imputed datasets, χ²(12) = 47.66-50.90, p < 0.001. Model fit explained 42.5-57.7% of variance (Cox & Snell R²) and 57.1-78.0% (Nagelkerke R²). Sensitivity and specificity in the original dataset were 92.0% and 88.2%, respectively. Across imputations, sensitivity ranged from 76.2 to 92.0% and specificity from 78.7 to 85.1%. Positive predictive values were 75.0-80.0%, and negative predictive values were 82.1-84.4%.

The results of the logistic regression analysis are summarised in Table 1. Two predictors were statistically significant. Referral after abnormal SFH measurement increased the odds of antenatal detection 154-fold (95% CI: 14.9-1603.4), while maternal smoking increased the odds of detection 3.9-fold (95% CI: 1.01-14.9).

**Table 1:** Logistic regression analysis of predictors of antenatal detection of small-for-gestational age (SGA) infants. Results from a multivariable (binary) logistic regression are presented. Odds ratios (OR) with 95% confidence intervals (CI) are shown. Analyses were run using IBM SPSS Statistics, version 26. A p-value < 0.05 was considered statistically significant. Statistically significant predictors are in bold. Trisomy screening risk (1) and (2) denote the two screening categories recorded in the BadgerNet database. OR: odds ratio; CI: confidence interval; BMI: body mass index (kg/m^2^); SFH: symphyseal fundal height; SGA: small-for-gestational age.

Predictor Variable	p-value	Odds Ratio (OR)	95% Confidence Intervals (CI)	
			Lower	Upper
Maternal age (years)	0.876	0.99	0.88	1.11
Body mass index (BMI, kg/m^2^)	0.555	0.97	0.87	1.08
Aspirin use	0.208	0.42	0.11	1.61
Previous small-for-gestational age (SGA) infant	0.552	0.67	0.18	2.53
Maternal smoking	0.049	3.87	1.01	14.93
Trisomy screening risk (1)	0.405	0.37	0.04	3.79
Trisomy screening risk (2)	0.446	0.55	0.12	2.56
Maternal ethnicity	0.282	0.73	0.42	1.30
Maternal hypertension	0.154	0.13	0.01	2.14
Maternal parity	0.153	0.58	0.27	1.23
Abnormal symphyseal fundal height (SFH) measurement	<0.001	154.4	14.9	1603.4
Maternal diabetes	0.760	2.47	0.01	807.7

Model performance

**Figure 2:**
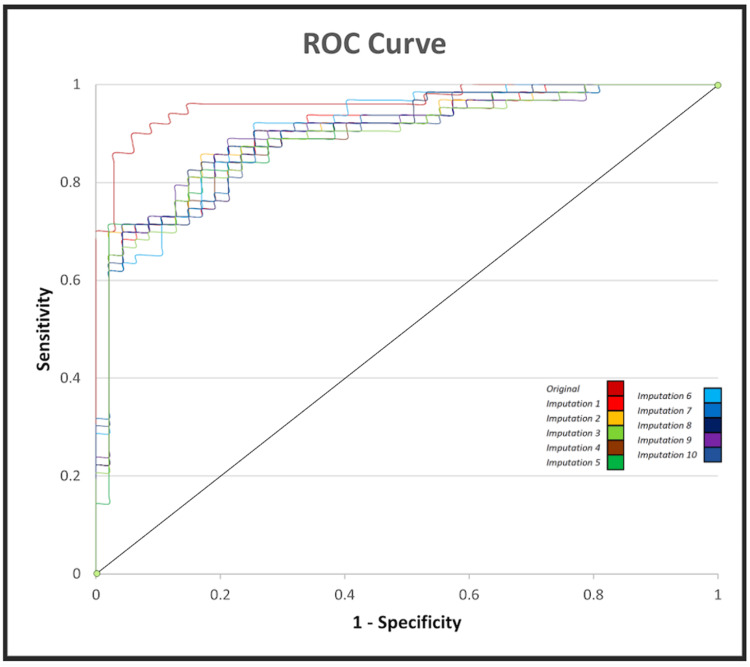
Receiver operating characteristic (ROC) curves for logistic regression model performance. ROC curves are shown for the original dataset and for each of the 10 imputed datasets. Discrimination was excellent with area under the curve (AUC) ranging from 0.89 to 0.91 across imputed datasets and was 0.96 for the original dataset. ROC analyses were performed using IBM SPSS Statistics, version 26. ROC: receiver operating characteristic; AUC: area under the curve.

Receiver operating characteristic (ROC) curves were generated using the predicted probabilities produced by our logistic regression model. As shown in Figure 2, ROC analysis demonstrated excellent model discrimination [19]. The area under the curve (AUC) was 0.96 for the original dataset and ranged from 0.89 to 0.91 for imputed datasets.

Supplementary analysis

To compare population-based with customised definitions, 109 infants were entered into GROW calculators. Of these, 105 were also classified as SGA, yielding a customised centile detection rate of 41.5%. Linear regression demonstrated that maternal smoking was significantly associated with lower customised centiles, F(1,107) = 10.48, p = 0.002. No significant association was observed between customised centiles and referral after abnormal SFH, F(1,107) = 1.62, p = 0.206.

## Discussion

Principal findings

In this service evaluation, the antenatal detection rate of SGA infants at MYHT was 42.7%, closely matching the national average of 42% reported among GAP users, although the top 10 performing units achieved a higher average detection rate of 56% [20]. This demonstrates compliance with national standards but highlights the need to aspire to best practice levels. Importantly, most SGA infants remain undetected: 67.2% at MYHT, 68% nationally, and 44% even at the best-performing sites [14,20].

Detection at MYHT was almost entirely reliant on referral following abnormal SFH measurements, with affected infants 154 times more likely to be identified antenatally. This finding supports SFH as a valuable routine screening tool [3,21]. Previous studies report a sensitivity of 27% and a specificity of 88% [22]. While this sensitivity is modest, SFH measurement remains an important first-line screening method when combined with serial plotting and follow-up ultrasound, as recommended by RCOG and NICE guidelines. However, reliance on SFH alone may indicate underutilisation of other risk-factor-based screening approaches. This raises important questions about balancing the benefits of wider case finding against the potential harms of unnecessary intervention, as highlighted in Saving Babies’ Lives, Version 2 [23].

Maternal smoking was the second independent predictor of antenatal detection, with smoking mothers being 3.9 times more likely to have an SGA infant identified. The association between smoking and reduced fetal growth is well established [24,25], with dose-dependent effects between tobacco use and reduced birthweight [26]. This higher detection rate likely reflects more severe growth restriction and/or closer surveillance. Our supplementary analysis demonstrated significantly lower customised birthweight centiles among infants of smoking mothers. Interestingly, smoking and non-smoking mothers were referred at similar rates following abnormal SFH measurements; however, infants of smoking mothers were still more likely to be identified. This may reflect more pronounced growth restriction in these pregnancies, increasing the likelihood of detection through ultrasound biometry.

Strengths and limitations

This study has several notable strengths, including the use of a large electronic maternity database over the study period, thus minimising selection bias and increasing generalisability to comparable healthcare settings. Additionally, robust statistical methods were used. This includes logistic regression with formally tested assumptions, multicollinearity was addressed, and multiple imputation was used to minimise bias from missing data. Data quality was further strengthened by cross-check extraction by two independent investigators. Finally, supplementary analyses compared population versus customised centiles, offering additional clinical insight and supporting the ongoing debate regarding optimal approaches to growth assessment.

This study also has limitations. Stillbirth cases were excluded, even though preventing stillbirth is a primary motivation for SGA surveillance. Missing data, which is inherent to the retrospective design, represented another limitation; however multiple imputation was used to provide unbiased estimates and preserve statistical power, avoiding the risks of listwise deletion [27,28]. As with all studies based on routinely collected electronic health records, there is potential for data entry errors and misclassification bias, particularly in variables such as smoking status and referral coding. Another limitation was reliance on BadgerNet’s automated population-based centile ranges (0.4-2, 2-9, 9-25). These bands exclude infants below the 0.4th centile and between the 9th and 10th centiles. When compared with GROW customised centiles, there was strong overlap with only four infants misclassified. However, some customised-defined SGA cases may have been missed entirely, since the initial dataset could only be generated using population-based centiles.

Further limitations include the single-centre design, relatively small sample size, and short study period. As the dataset covered only three months in early 2020, external influences such as seasonal variation and service pressures from the emerging COVID-19 pandemic may have affected referral patterns and detection rates. These factors may limit generalisability to other settings and time periods. Reliance on routinely collected clinical data also introduces the possibility of recording bias. External validation or calibration analyses were not feasible within the scope of this single-site service evaluation due to data governance restrictions and sample size, but future multicentre work is recommended to confirm these findings in broader NHS contexts. Together these factors may limit generalisability of the findings.

Clinical implications and future directions

This evaluation reinforces the central role of SFH referral and maternal smoking status in SGA detection. However, the heavy reliance on SFH highlights the need to optimise other elements of antenatal surveillance, particularly risk-factor-based screening. Despite compliance with national averages, the majority of cases remain undetected, underscoring the importance of aiming for best-practice performance as achieved by the top 10 units.

The use of population-based centiles within BadgerNet is a key limitation of current surveillance. We recommend that customised GROW centiles be automatically integrated into BadgerNet. As MYHT is a GAP registered Trust, this would facilitate consistent evaluation and allow benchmarking against other GAP sites. Additionally, abandoning centile bands in favour of exact centiles would increase granularity, improve accuracy, and reduce misclassification.

Equity of care is also an important consideration. The stronger association between smoking and detection may reflect closer clinical monitoring in high-risk groups, but it raises concerns that pregnancies without obvious risk factors may receive less surveillance. Ensuring consistent and systematic application of screening across all groups is therefore crucial.

Finally, smoking cessation in pregnancy remains a cornerstone of prevention. Reducing smoking prevalence would not only lower the incidence of SGA but may also enhance detection by narrowing the overlap between constitutionally small and growth-restricted infants. Future prospective, multicentre studies are required to evaluate whether integration of customised centiles and strengthened public health measures translates into improved detection rates and perinatal outcomes.

## Conclusions

This service evaluation highlights the key role of SFH measurement in detecting SGA infants. Routine screening, including SFH assessment and risk profiling (e.g., smoking status), remains vital for identifying SGA and FGR. The reliance on SFH underscores the need for standardised risk-factor pathways and integration of customised GROW centiles within BadgerNet. SGA and FGR are major contributors to perinatal morbidity and mortality, and antenatal detection is crucial to reducing these risks; however, detection remains variable. At MYHT, the 42.7% detection rate matches the national average, but average performance still means most cases are missed. The goal should be to match the higher detection rates achieved by leading units.

Once SGA is suspected, optimised antenatal care is essential, including regular ultrasound scans, obstetric review, and individualised pregnancy and delivery planning. Future work should examine not only detection rates but whether earlier identification improves management quality and perinatal outcomes.
